# The transcription factor *Pax6* is required for pancreatic β cell identity, glucose-regulated ATP synthesis, and Ca^2+^ dynamics in adult mice

**DOI:** 10.1074/jbc.M117.784629

**Published:** 2017-04-04

**Authors:** Ryan K. Mitchell, Marie-Sophie Nguyen-Tu, Pauline Chabosseau, Rebecca M. Callingham, Timothy J. Pullen, Rebecca Cheung, Isabelle Leclerc, David J. Hodson, Guy A. Rutter

**Affiliations:** From the ‡ Section of Cell Biology and Functional Genomics, Division of Diabetes, Endocrinology, and Metabolism, Imperial College London, Du Cane Road, London W12 0NN, United Kingdom,; the § Institute of Metabolism and Systems Research and Centre of Membrane Proteins and Receptors, University of Birmingham, Edgbaston B15 2TT, United Kingdom, and; the ¶ Centre for Endocrinology, Diabetes, and Metabolism, Birmingham Health Partners, Birmingham B15 2TH, United Kingdom

**Keywords:** calcium, diabetes, gene expression, imaging, islet, insulin

## Abstract

Heterozygous mutations in the human paired box gene *PAX6* lead to impaired glucose tolerance. Although embryonic deletion of the *Pax6* gene in mice leads to loss of most pancreatic islet cell types, the functional consequences of *Pax6* loss in adults are poorly defined. Here we developed a mouse line in which *Pax6* was selectively inactivated in β cells by crossing animals with floxed *Pax6* alleles to mice expressing the inducible Pdx1CreERT transgene. *Pax6* deficiency, achieved by tamoxifen injection, caused progressive hyperglycemia. Although β cell mass was preserved 8 days post-injection, total insulin content and insulin:chromogranin A immunoreactivity were reduced by ∼60%, and glucose-stimulated insulin secretion was eliminated. RNA sequencing and quantitative real-time PCR analyses revealed that, although the expression of key β cell genes, including *Ins2*, *Slc30a8*, *MafA, Slc2a2*, *G6pc2*, and *Glp1r*, was reduced after *Pax6* deletion, that of several genes that are usually selectively repressed (“disallowed”) in β cells, including *Slc16a1*, was increased. Assessed in intact islets, glucose-induced ATP:ADP increases were significantly reduced (*p* < 0.05) in β*Pax6*KO *versus* control β cells, and the former displayed attenuated increases in cytosolic Ca^2+^. Unexpectedly, glucose-induced increases in intercellular connectivity were enhanced after *Pax6* deletion, consistent with increases in the expression of the glucose sensor glucokinase, but decreases in that of two transcription factors usually expressed in fully differentiated β-cells, *Pdx1* and *Nkx6.1*, were observed in islet “hub” cells. These results indicate that *Pax6* is required for the functional identity of adult β cells. Furthermore, deficiencies in β cell glucose sensing are likely to contribute to defective insulin secretion in human carriers of *PAX6* mutations.

## Introduction

Defective insulin secretion underlies all forms of diabetes mellitus, a disease that now affects more than 422 million individuals worldwide (World Health Organization). Insulin is stored and released from β cells within pancreatic islets of Langerhans following elevations in glucose concentration (1). In response to high glucose, enhanced flux through glycolysis and mitochondrial oxidative metabolism (2) leads to increases in cytosolic ATP/ADP concentrations, the closure of ATP-sensitive K^+^ (K_ATP_) channels (3), cellular depolarization, and Ca^2+^ influx (4). Ca^2+^ sensors on secretory granules then catalyze hormone release through exocytosis (5). In the context of the intact islet, β cell-β cell connections (6, 7) then create a coordinated network ensuring the optimal regulation of secretion through propagation of Ca^2+^ and other signals (8).

Paired box 6 (PAX6) is a transcription factor crucial for the development of the eye, brain, olfactory system, and endocrine pancreas. Heterozygous mutations in the *PAX6* gene, which result in the production of a truncated, non-functional protein (9), cause abnormal iris formation (aniridia) and impaired glucose tolerance (10). Correspondingly, PAX6 binding domains are found in the promoter regions of several key β cell genes (11), and islets derived from a human pedigree harboring an inactivating missense *PAX6* mutation are deficient in proinsulin processing enzymes (PCSK1/3) (12). Interestingly, we observed no changes in *Pcsk1* expression in this study, arguing that the alterations observed in man may reflect an indirect action of PAX6. Furthermore, inheritance of the G allele at the single nucleotide polymorphism rs685428 lowers *PAX6* expression in man and is associated with increased fasting insulin and lower proinsulin:insulin ratio (13).

In mice, homozygocity for the “small eye” *Pax6* mutant allele (Sey^Neu^) leads to death at perinatal stages, and affected animals have dramatically reduced numbers of all islet cell types (14). Although deletion throughout the pancreas leads to overt diabetes and loss of β cells (15), heterozygous loss-of-function mutants show age-dependent (12) and diet-dependent (16) impairments in glucose tolerance. Finally, *Pax6* expression is decreased in a rat model of T2D (the Zucker diabetic fatty rat) (17).

Recent studies have also indicted that PAX6 may be important in maintaining the differentiated state and identity of the adult β cell. Thus, conditional inactivation of *Pax6* at post-natal stages in mice with a tamoxifen-inducible ubiquitous *Cre* leads to the development of a severe diabetic phenotype (18). Pancreatic analysis revealed a reduction in the expression of the *insulin 1* and *insulin 2*, *glucagon*, and *somatostatin* genes, coupled with increases in the number of ghrelin-positive cells (18). The latter were also increased when deletion was restricted to either α or β cells in adult mice (19).

By contrast, few studies have examined how *Pax6* deletion affects the functional maturity of the adult β cell. One report (20), based on RNA interference, provided evidence that *Pax6* is required in the adult rat β cell for normal insulin secretion and the expression of key genes, including *Ins1* and *Ins2*. However, the above approach was complicated by the requirement for β cell isolation prior to gene silencing and by potential off-target effects of silencing RNAs. Further, glucose sensing was examined in the latter study using isolated β cells rather than the intact islet, where responses to stimulation can differ markedly, and the gain of function resulting from intercellular interactions across the islet syncytium is lost (21). On the other hand, studies on the effect of *Pax6* deletion using *Cre*-mediated recombination in the adult have used either a ubiquitous *Cre* (18) or *Cre* expression driven by the rat insulin 2 promoter (RIP), which also leads to substantial recombination in the brain (22).

The aims of the present work were therefore to achieve efficient *Pax6* deletion selectively in the adult mouse β cell using targeted recombination at floxed alleles with an alternative tamoxifen-inducible *Cre* system (Pdx1CreERT) (23, 24), to examine β cell function and glucose-sensing *in vivo* and *in vitro* after *Pax6* ablation, and to determine the role of PAX6 in the control of a broader range of genes than what has been examined previously, including those that are normally selectively silenced, or “disallowed,” in mature β cells (25, 26). Alongside decreases in the expression of β cell signature genes, up-regulation of the latter, which occurs in type 2 diabetes (27, 28), is likely to report a loss of normal cellular identity.

We show that *Pax6* deletion achieved in this way leads to profound diabetes, consistent with earlier findings using alternative *Cre* drivers. Critically, we demonstrate marked abnormalities in gene expression, glucose metabolism, Ca^2+^ dynamics, and insulin secretion in β*Pax6*KO mouse islets early in the development of diabetes, which are likely to play an important part in β cell secretory failure. Unexpectedly, islets null for *Pax6* in the β cell display normal or enhanced cellular interconnectivity. Thus a functionally interconnected β cell network can be maintained despite the partial loss of full β cell identity.

## Results

### Efficient and inducible deletion of Pax6 from the adult mouse β cell

**Figure 1.**
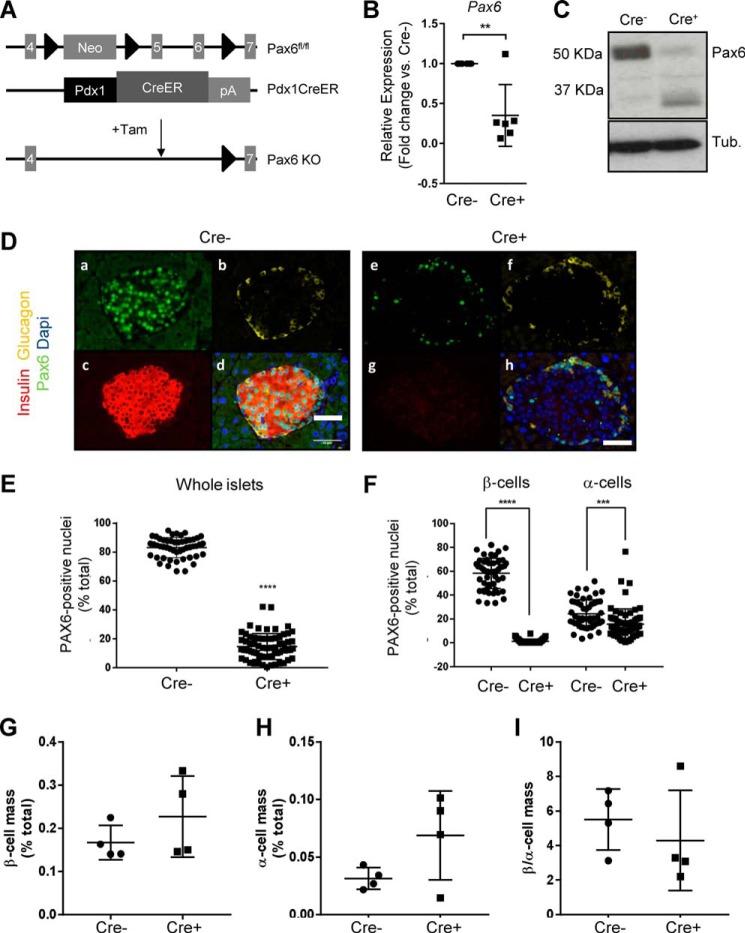
**Inducible deletion of *Pax6* from pancreatic β cells does not affect β-cell mass.** *A*, gene deletion was caused by tamoxifen (*Tam*)-induced Pdx1CreER-mediated removal of exons 5, 5a, and 6 of *Pax6. B* and *C*, qRT-PCR (*B*; **, *p* < 0.01; Student's *t* test; *n* = 5 each genotype; values represent mean ± S.D.) and Western blot analysis of islets isolated from β*Pax6*KO (*Cre*^+^) and litter mate controls (*Cre*-) mice (*C*). Note the presence of a lower-molecular-mass band in the Cre^+^ case, reflecting the likely generation of a truncated Pax6 protein lacking exons 5 and 6. *Tub*, tubulin. *D–F*, immunocytochemical analysis was performed as given under “Experimental Procedures.” Pancreatic slices from wild-type controls (*a–d*) and PAX6 knock-out mice (*e–h*) were stained for PAX6 (1:70), insulin (1:200), and glucagon (1:500) (*scale bars* = 50 μm). Images show staining of PAX6 (*a* and *e*), glucagon (*b* and *f*) and insulin (*c* and *g*). All channels were overlaid into a composite image with DAPI staining to visualize nuclear staining (*d* and *h*). *E*, the percentage of PAX6-positive nuclei was significantly decreased in βPax6KO in whole-islet cell populations compared with littermate controls. (****, *p* < 0.0001; Student's *t* test; *n* = 4 each genotype). *F*, the percentage of PAX6-positive nuclei in β cells was significantly decreased in βPax6KO mice. (****, *p* < 0.0001; Student's *t* test; *n* = 5 each genotype). A significant but smaller reduction in PAX6-positive nuclei was present in α cells. Values represent mean ± S.D. *G* and *H*, β cell mass and α cell mass represent the percentage of respectively insulin-positive (*G*) and glucagon-positive (*H*) staining area over the total pancreas section using ImageJ software. *I*, the β to α cell ratio was obtained by measuring the total insulin/glucagon-positive area on each pancreas section. A total of five pancreas sections, evenly separated by 25 μm, from each animal and genotype were used (*n* = 4 animals in each group, *p* = ns by Student's *t* test).

Mice harboring *Lox*P sites surrounding exons 5, 5a, and 6 of *Pax6* were crossed to Pdx1CreER mice. The breeding strategy used resulted in all animals carrying two copies of the floxed *Pax6* gene, but only half of these animals possessed a *Cre* allele (*Cre*^+^; *Pax6*^fl/fl^::Pdx1CreERT^+/−^; β*Pax6*KO). Tamoxifen is expected to result in gene deletion only in animals that possess the *Cre* transgene (Fig. 1*A*).

Eight week-old *Pax6*^fl/fl^::Pdx1CreERT^+/−^ and control *Pax6*^fl/fl^:: Pdx1CreERT^−/−^ littermates were injected daily with tamoxifen (2 mg) for 5 days. Islets isolated 2 weeks after the final tamoxifen injection showed substantially (∼70%) reduced levels of *Pax6* mRNA compared with littermate controls (Fig. 1*B*, 1.00 ± 0.00 *versus* 0.35 ± 0.16 for *Cre*^−^ *versus Cre*^+^, -fold change *versus Cre*^−^, *p* < 0.01), consistent with deletion from the majority of the islet β cell compartment (∼60% of all cells) (29). A comparable reduction in PAX6 immunoreactivity was also observed by Western blotting (Fig. 1*C*).

Immunocytochemical analysis of pancreatic slices for PAX6, insulin, and glucagon assessed 8 days after the first tamoxifen injection (Fig. 1*D*) revealed an ∼80% decrease in PAX6 staining across all islet cells (Fig. 1, *D* and *E*) with essentially complete (>98%) elimination of PAX6 immunoreactivity from β-cells and a more minor reduction (∼25%) in α cells (Fig. 1, *D* and *F*), presumably reflecting maintained expression of *Pdx1* in a minority of α cells, some of which may also express other islet hormones (30). We note that this *Cre* deleter strain does not catalyze significant recombination in the hypothalamus (31).

Despite a dramatic decrease in insulin immunoreactivity on a per-cell basis (Fig. 1*D*, *g versus c*), immunocytochemical analysis (Fig. 1*G*), assessed at the same time point, revealed no significant changes in total β cell mass (Fig. 1*G*, 0.23 ± 0.05% *versus* control 0.17 ± 0.02%, ns,^4^ Student's *t* test, *n* = 4 animals/genotype), α cell mass (Fig. 1*H*, 0.069 ± 0.019% *versus* control 0.031 ± 0.004%, ns, Student's *t* test, *n* = 4) or β to α cell ratio (Fig. 1*I*, 4.3 ± 1.5 *versus* control 5.5 ± 0.9, ns, Student's *t* test, *n* = 4).

### Deletion of Pax6 impairs glucose homeostasis in vivo

**Figure 2.**
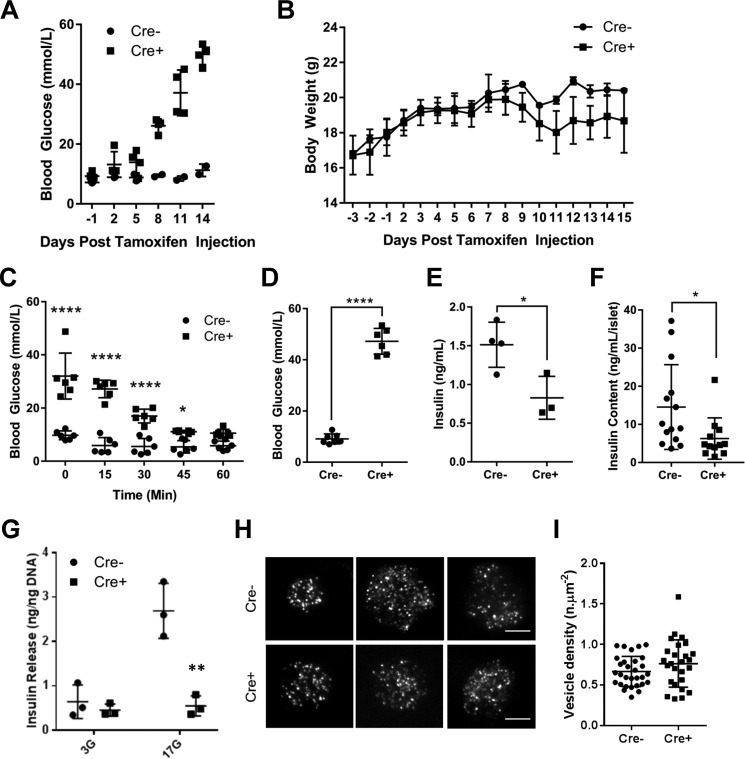
**Deletion of *Pax6* in the β cell causes severe hyperglycemia and reduces insulin levels.** *A* and *B*, fed glycemic profile (*A*) and accompanying body weight (*B*) of β*Pax6KO* and littermate control mice (*n* = 2 *Cre*^−^ and 4 *Cre*^+^ mice). *C*, challenging mice with a 0.75 units/kg body weight insulin injection showed normal insulin sensitivity despite severe hyperglycemia (*, *p* < 0.05; ****, *p* < 0.0001; two-way analysis of variance; *n* = 6 animals each genotype). *D* and *E*, upon termination 2 weeks post-tamoxifen treatment, glucose levels were increased (*D*; ****, *p* < 0.0001; Student's *t* test; *n* = 7 *Cre*^−^ and 6 *Cre*^+^ mice) coupled with decreased insulin levels (*E*; *, *p* < 0.05; Student's *t* test; *n* = 4 *Cre*^−^ and 3 *Cre*^+^ mice). *F*, five islets were lysed in acidified ethanol, and insulin content was determined by HTRF assay (*, *p* < 0.05; Student's *t* test; *n* = 14 *Cre*^−^ and 12 *Cre*^+^ replicates). *G*, insulin secretion from isolated islets (see “Experimental Procedures” for details). **, *p* < 0.05 by Student's *t* test for the effect of Pax6 deletion. *H*, TIRF analysis of granule distribution involving *n* = 30 separate cells for control and *n* = 26 for null mouse islets. *Scale bars*, 5 μm. *I*, analysis of data obtained as in *H*. Values represent mean ± S.D.

β*Pax6* KO mice showed increased blood glucose levels from 8 days after final tamoxifen injection (Fig. 2*A*, 9.45 ± 0.35 mm *versus* 26.15 ± 1.13 mm for *Cre*^−^ *versus Cre*^+^, respectively). This change was further exacerbated over time, with β*Pax6*KO animals showing an average glycemia of 50.0 mm at the time of termination. Consistent with overt diabetes, the body weight of β*Pax6*KO mice began to decline 7 days after the final tamoxifen injection (Fig. 2*B*). Two weeks after tamoxifen treatment, the decline in body weight was so severe that β*Pax6*KO mice were terminated in accordance with United Kingdom Home Office guidelines. We note that expression of the Pdx1CreERT transgene alone is not expected to impact insulin secretion or glucose tolerance (32). Correspondingly, *Pax6*^fl/fl^::Pdx1CreERT^+/−^ mice remained normoglycemic in the absence of tamoxifen administration (data not shown). Likewise, tamoxifen alone, at the doses used here, exerts no effect on glycemia in wild-type C57BL/6J mice (31). Hence, any metabolic changes observed can reasonably be ascribed to the deletion of *Pax6*.

Insulin sensitivity, as assessed by intraperitoneal insulin tolerance test, was not markedly affected by *Pax6* deletion in β cells despite increased blood glucose levels (Fig. 2*C*). However, upon termination 2 weeks after tamoxifen treatment, blood glucose levels were significantly higher in β*Pax6*KO animals than in littermate controls (Fig. 2*D*, 9.10 ± 0.14 mm *versus* 47.2 ± 0.04 mm for *Cre*^−^ *versus Cre*^+^, *p* < 0.001). This change was accompanied by significantly decreased plasma insulin levels (Fig. 2*E*, 1.51 ± 0.15 ng/ml *versus* 0.83 ± 0.16 ng/ml for *Cre*^−^ *versus Cre*^+^, *p* < 0.05), suggesting that the observed hyperglycemia, caused by *Pax6* deletion, is likely to be due to insufficient insulin output from pancreatic β cells.

Total insulin levels, as measured in islets isolated from animals 1 week after tamoxifen treatment, were significantly reduced by *Pax6* deletion (Fig. 2*F*; 14.5 ± 2.97 ng/ml/islet *versus* 6.28 ± 1.57 ng/ml/islet for *Cre*^−^ *versus Cre*^+^, respectively; *p* < 0.05). A similar reduction, to levels observed after incubation at 3 mm glucose, was apparent after normalization to islet DNA content (660 ± 63 *versus* 322 ± 69 ng insulin/ng DNA, *p* < 0.05) or to total insulin content (0.41 ± 0.03 *versus* 0.016 ± 0.03%, *p* < 0.01). The latter observation excludes the possibility that our failure to detect an increase in insulin secretion from β*Pax6*KO islets after incubation at 17 *versus* 3 mm glucose simply reflects the lowered insulin content of KO *versus* control islets.

Finally, we measured basal and glucose-stimulated insulin secretion from isolated islets (Fig. 2*G*). Release of insulin from β*Pax6*KO islets tended to be lower than that from control islets when measured at 3 mm glucose, but, in contrast to the ∼4-fold stimulation observed in wild-type islets at 17 mm glucose, no further increase was detectable from β*Pax6*KO islets at this higher glucose concentration (Fig. 2*G*). This altered response was not associated with any marked redistribution of granules away from the plasma membrane (*i.e.* in a reduction in the number of “morphologically docked” granules), as assessed by TIRF microscopy (Fig. 2, *H* and *I*), suggesting that defects later in granule release, or in the generation of metabolic signals required for exocytosis, are defective in this model.

### Deletion of Pax6 alters the expression of β cell signature and disallowed genes

**Figure 3.**
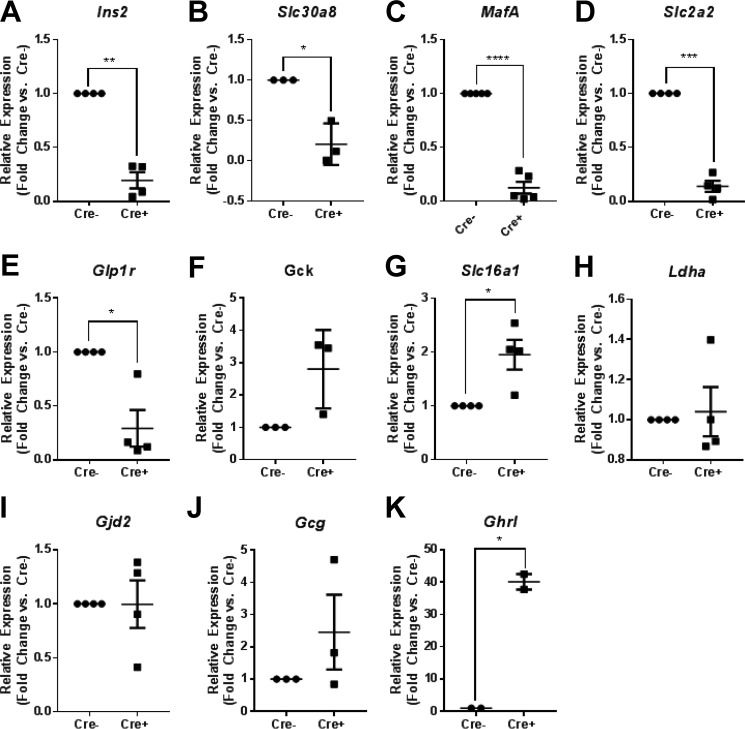
**Islet gene expression is altered by *Pax6* deletion.** RNA was extracted from islets isolated from β*Pax6*KO and littermate control mice and reverse-transcribed before assessment of gene expression. *A–K*, expression of *Ins2* (*A*; **, *p* < 0.01), *Slc30a8* (*B*; *, *p* < 0.05), *Mafa* (*C*; ****, *p* < 0.0001), *Slc2a2* (*D*, ***, *p* < 0.001), *Glp1r* (*E*; *, *p* < 0.05), *Gck* (*F*, ns), *Slc16a1* (*G*, *p < 0.05), *Ldha* (*H*, ns), *Gjd2* (*I*, ns), *Gcg* (*J*, ns), and *Ghrl* (*K*, *p < 0.05; all paired Student's *t* test *versus Cre*^−^; *n* = 3–4 each genotype) was assessed in both β*Pax6*KO and littermate control mice. Values represent mean ± S.D. *L*, GSEA for disallowed genes (see “Results”). *M* and *N*, genes with increased (*green*) or decreased (*red*) expression in β*Pax6*KO *versus* control mice based on RNAseq analysis.

Consistent with a loss of β cell identity, the expression of *Ins2*, *Slc30a8*, *MafA, Slc2a2*, and *Glp1r,* signature genes, which are all highly expressed in healthy β cells and are essential for normal function (1), were all significantly reduced by *Pax6* deletion 8 days after tamoxifen treatment, as assessed by qRT-PCR analysis (Fig. 3, *A–E*; *p* < 0.05 *versus Cre-* for all genes). In contrast, the expression of the glucose sensor glucokinase (*Gck*) tended to increase (Fig. 3*F*, ns *versus Cre*^−^). Interestingly, the expression of the β cell disallowed gene *Slc16a1*, encoding monocarboxylate transporter 1 (MCT-1), was significantly increased by *Pax6* deletion (Fig. 3*G*, *p* < 0.05 *versus Cre*^−^) at this stage. However, the expression of *Ldha*, another disallowed gene, was not affected (Fig. 3*H*, *p* > 0.05 *versus Cre*^−^).

As expected based on earlier models (18, 19), glucagon expression was not significantly altered by *Pax6* deletion from β cells (Fig. 3*J*, ns *versus Cre*^−^). Correspondingly, fasting plasma glucagon levels were also unchanged between control and *Pax6* null animals (147 ± 49.6 and 128 ± 28.7 pg/ml in control and β*Pax6*KO mice; *n* = 4 and 6 animals, respectively; *p* > 0.05). In contrast, ghrelin expression was dramatically (>40-fold) increased (Fig. 3*K*, *p* < 0.05 *versus Cre*^−^).

To provide a transcriptome-wide assessment of the impact of *Pax6* deletion, we next performed massive parallel RNA sequencing (RNAseq) on islets from control or null animals (supplemental Fig. S1). This revealed, first, that disallowed genes were selectively enriched among the up-regulated genes from β*Pax6*KO islets, as assessed by GSEA (supplemental Fig. S1*A*). Thus, of 58 members of the disallowed gene family included in the analysis (25), 38 were significantly enriched. These included both founder members of the group (*Slc16a1* and *Ldha*) (33) as well as the four other members (*Pdgfra*, *Cxcl12*, *Igfbp4*, and *Oat*) of the shorter list of six genes common to Refs. 25 and 26 as given in the histogram in supplemental Fig. S1*B*.

RNAseq also demonstrated that, although markers of β cell progenitors, notably Neurogenin3, were up-regulated in response to the loss of PAX6, transcription factors characteristic of the fully differentiated β cell, including *Nkx6.1*, *Pdx1*, and *MafA*, were repressed in knock-out islets (supplemental Fig. S1*C*). Interestingly, these observations suggest that PAX6 may serve as either an activator or a repressor depending on context and target, as shown recently for the β cell-enriched factors RFX6 (34) and Nkx2.2 (35).

The above approach also corroborated the changes observed above, including in the expression of *ghrelin* (increased 32-fold), *Ins2* (decreased 5.5-fold), and *Gck* (increased 2.8-fold) after *Pax6* deletion. *Gcg*, *Ppy*, and *SSt* expression was barely altered (supplemental Fig. S1*D*), and *Pcsk1* and *Pcsk2* mRNA levels were essentially unchanged (supplemental Fig. S1*E*), arguing against alterations in prohormone processing. Affected genes included *Ero1lb*, a protein disulfide isomerize strongly enriched in β cells and likely involved in insulin maturation (36), and *G6pc2*, involved in glucose sensing (37), each being significantly down-regulated (supplemental Fig. S1*E*). Of note, we observed a large (156-fold, *p* = 1.5 × 10^−215^) increase in the expression of the voltage-gated potassium channel *Kcnj5*, likely to exert significant effects on membrane excitability and thus Ca^2+^ dynamics (supplemental Fig. S1*E*).

### βPax6KO islet cells lose insulin expression but retain neuroendocrine properties

**Figure 4.**
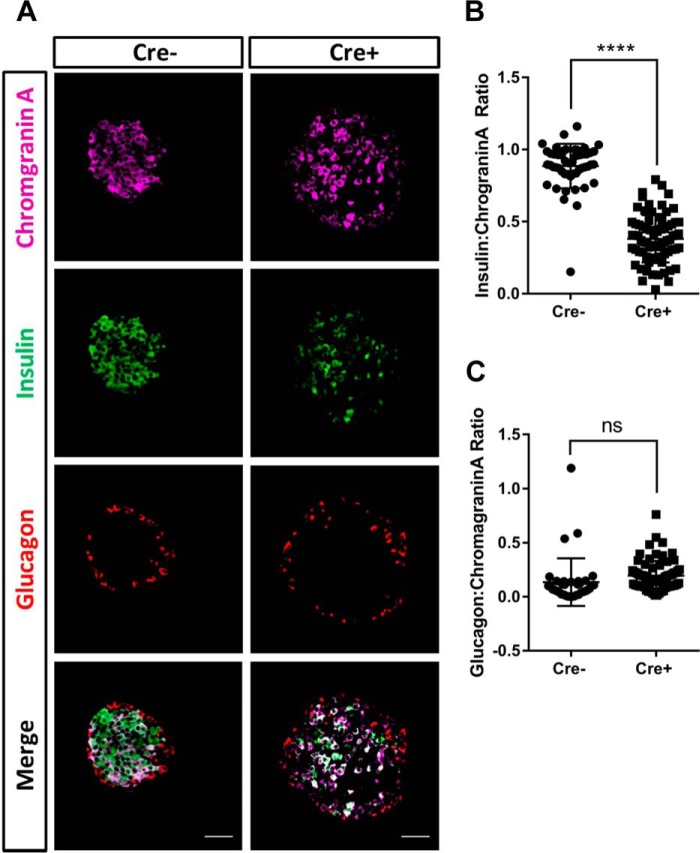
**β*Pax6*KO islets exhibit reduced insulin-positive cells but retain neuroendocrine properties.** *A*, pancreatic slices were stained for chromogranin A (1:200), insulin (1:200), and glucagon (1:1000). *Scale bar* = 50 μm. *B*, quantification of islet insulin:chromogranin A ratio (*p* < 0.001; Student's *t* test; *n* = 50 *Cre*^−^ and 75 *Cre*^+^ islets). *C*, as for *B*, but showing the glucagon:chromogranin A ratio (ns, Student's *t* test, *n* = 37 *Cre*^−^ and 70 *Cre*^+^ islets). Values represent mean ± S.D.

Previous reports (19) and the above findings indicate that *Pax6*-null β cells are shunted toward an alternative cell fate. We therefore stained pancreatic slices obtained from animals 8 days after the final tamoxifen injection for the neuroendocrine marker chromogranin A (38) alongside insulin and glucagon (Fig. 4*A*). Expressing the number of total insulin- and glucagon-immunopositive area as a ratio of the total chromogranin A-immunopositive area revealed that deletion of *Pax6* significantly reduced the insulin:chromogranin A ratio (Fig. 4*B*; 0.89 ± 0.02 *versus* 0.38 ± 0.02 for *Cre*^−^ *versus Cre*^+^, respectively; *p* < 0.001), whereas the glucagon:chromogranin A ratio was not significantly changed (Fig. 4*C*, 0.14 ± 0.04 *versus* 0.19 ± 0.02 for *Cre*^−^ *versus Cre*^+^, ns).

### Islets isolated from βPax6KO mice show impaired glucose-induced cytosolic ATP/ADP increases and intracellular Ca^2+^ dynamics

**Figure 5.**
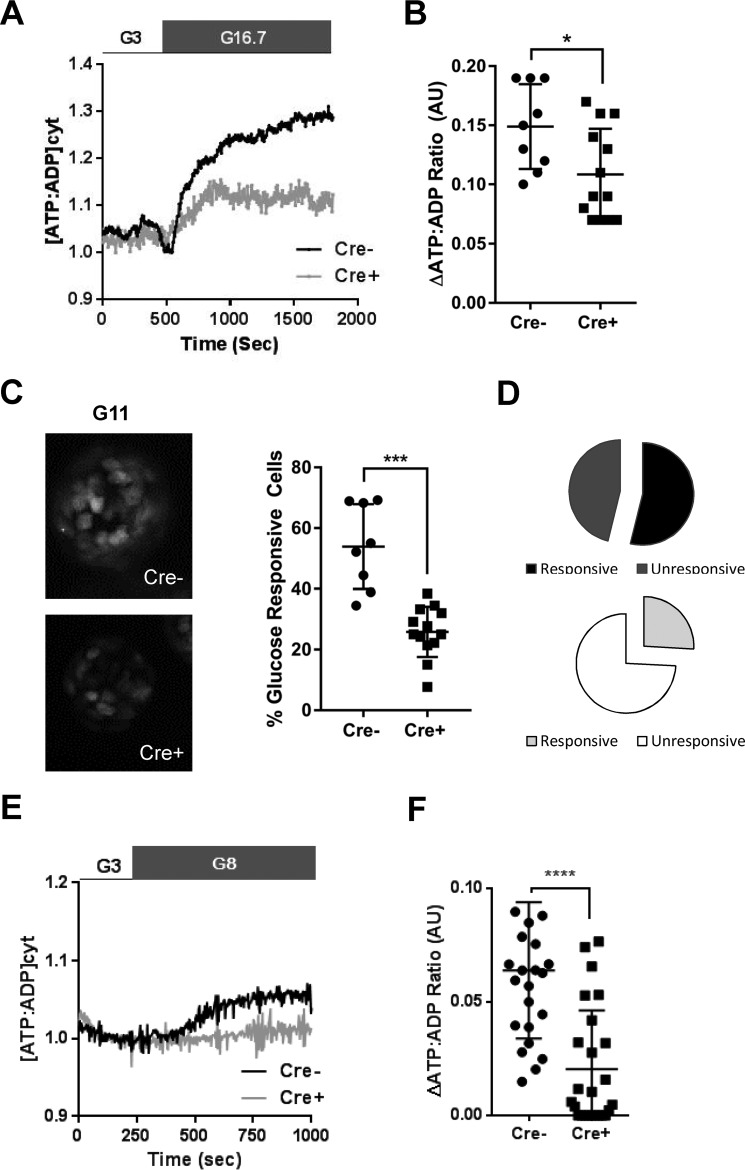
**Islets isolated from β*Pax6*KO mice display altered glucose-evoked ATP responses.** *A* and *B*, ATP rises in response to 11 mm glucose (*A*, representative traces) were reduced in β*Pax6*KO islets (*B*; *, *p* < 0.05; Student's *t* test; *n* = 9 *Cre*^−^ and 13 *Cre*^+^ islets). *C* and *D*, the number of cells that responded to 11 mm glucose was also substantially reduced (***, *p* < 0.001; Student's *t* test; *n* = 9 *Cre*^−^ and 13 *Cre*^+^ islets). *E* and *F*, similar findings were made using 8 mm glucose (****, *p* < 0.001; Student's *t* test; *n* = 25 *Cre*^−^ and 25 *Cre*^+^ islets). Values represent mean ± S.D.

Given the absence of glucose-stimulated insulin secretion and the changes in gene expression and apparent cellular identity observed above, we next determined whether disruption of *Pax6* may interfere with glucose metabolism or sensing by β cells within the intact islet. We first used the recombinant cytosol-targeted ATP/ADP probe Perceval (39, 40) and Nipkow spinning disc confocal microscopy (41, 42). Expression of the above sensor is largely restricted to the β cell (34) because of the apparent tropism of adenoviruses toward this cell type (43). Exposure to high (11 mm) glucose caused a time-dependent increase in ATP/ADP ratio in control islets as expected (40, 41), and this increase was reduced in amplitude by ∼30% in β*Pax6*KO islets (Fig. 5, *A* and *B*; 0.15 ± 0.01 arbitrary units (AU) *versus* 0.11 ± 0.01 AU for *Cre*^−^ *versus Cre*^+^, respectively; *p* < 0.05; supplemental Movies 1 and 2). *Pax6* deletion also markedly reduced the number of glucose-responsive cells, as assessed at 11 mm glucose (Fig. 5, *C* and *D*; 53.9 ± 4.93 *versus* 25.8 ± 2.30 for *Cre*^−^ and *Cre*^+^, respectively; *p* < 0.001). Similar differences between β*Pax6*-null and control islets were also apparent after stimulation at a lower glucose concentration (8 mm; Fig. 5, *E* and *F*; 0.07 ± 0.01 *versus* 0.02 ± 0.005 AU30 for *Cre*^−^ and *Cre*^+^, respectively; *p* < 0.001).

**Figure 6.**
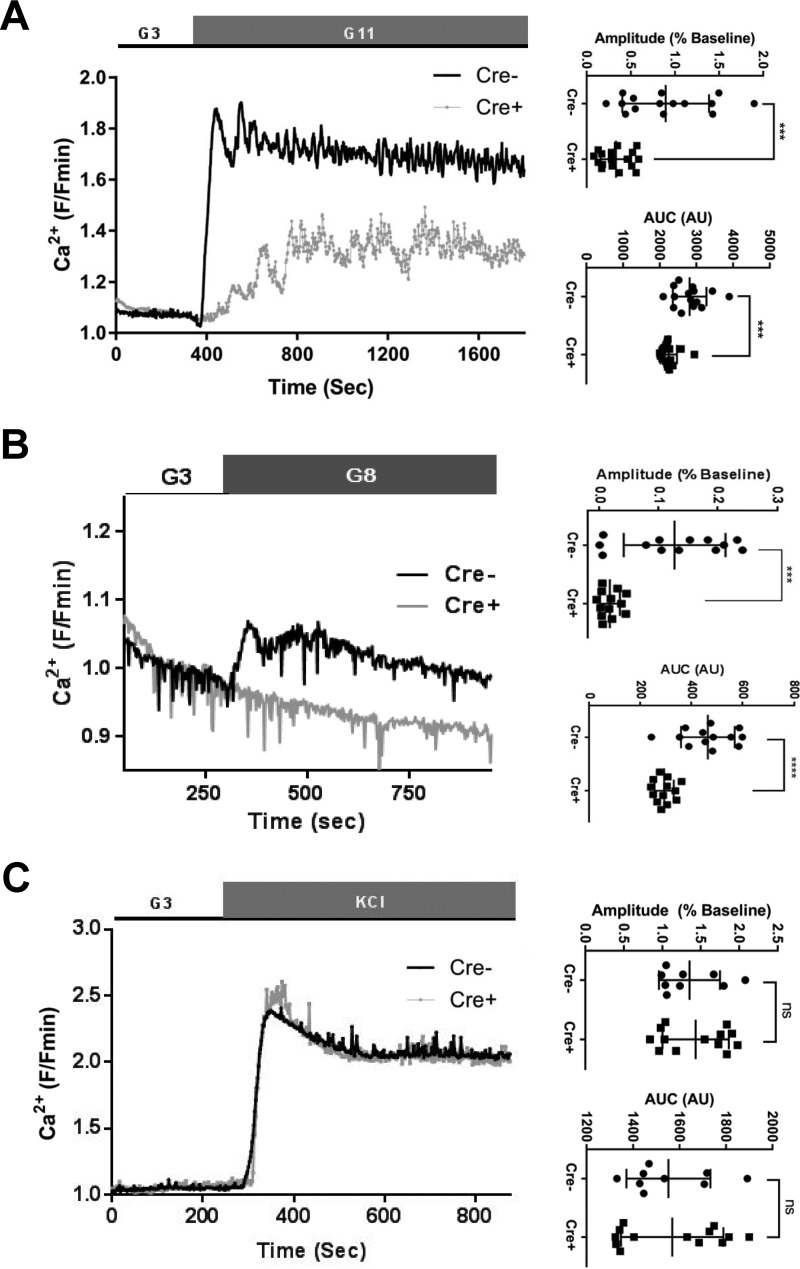
**β*Pax6KO* islets exhibit altered Ca^2+^ dynamics.** *A* and *B*, Nipkow spinning disk microscopy and the Ca^2+^ dye Fluo-2 were used to assess [Ca^2+^]*_i_* dynamics in response to 11 mm glucose (*A*; ***, *p* < 0.001; Student's *t* test; *n* = 15 *Cre*^−^ and 17 *Cre*^+^ islets) or to 8 mm glucose (*B*; <0.001; Student's *t* test; *n* = 13 *Cre*^−^ and 13 *Cre*^+^ islets). *C*, no differences were apparent in the response to depolarization with KCl (ns, Student's *t* test, *n* = 9 *Cre*^−^ and 13 *Cre*^+^ islets). Values represent mean ± S.D.

Because impairments in ATP generation are likely to affect the closure of K_ATP_ channels, impacting plasma membrane depolarization and Ca^2+^ influx (1), we next sought to determine how intracellular Ca^2+^ dynamics may be affected by *Pax6* deletion. Islets were therefore loaded with the trappable intracellular Ca^2+^ probe Fluo-2, and confocal imaging was performed essentially as above (albeit at a higher acquisition rate). *Pax6* deletion reduced both the amplitude (Fig. 6*A*; 0.90 ± 0.13 AU *versus* 0.33 ± 0.04 AU for *Cre*^−^ *versus Cre*^+^, respectively; *p* < 0.001) and the area under the curve (Fig. 6*A*, 2819.8.1 ± 118.1 AU *versus* 2248 ± 54.9 AU for *Cre*^−^ *versus Cre*^+^, *p* < 0.001) of glucose-evoked Ca^2+^ traces. Ca^2+^ responses to the depolarizing stimulus KCl were unchanged by *Pax6* deletion (Fig. 6*B*; amplitude, 1.35 ± 0.13 AU *versus*1.43 ± 0.12 AU for *Cre*^−^ *versus Cre*^+^, respectively; area under the curve, 1552 ± 60.2 *versus* 1569 ± 61.1 for *Cre*^−^ *versus Cre*^+^, respectively; both ns), suggesting that voltage-dependent Ca^2+^ channel function remains intact.

### Individual β cell Ca^2+^ responses in βPax6KO islets show high levels of coordination

**Figure 7.**
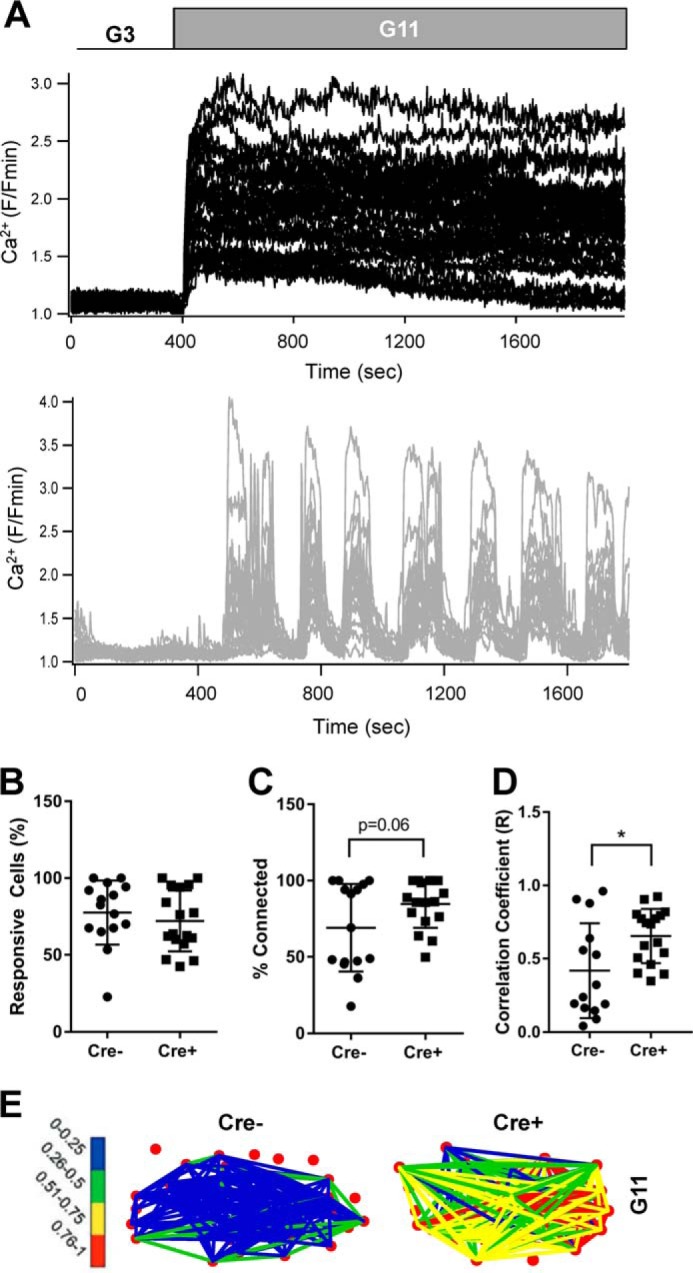
**Islet cell Ca^2+^ responses remain highly coordinated in β*Pax6KO* islets.** *A*, individual cellular Ca^2+^ responses from control (*top panel*) and β*Pax6KO* (*bottom panel*) islets. *B*, percentage of glucose-responsive islet cells (ns, Student's *t* test, *n* = 15 *Cre*^−^ and 17 *Cre*^+^ islets). *C–E*, connectivity analysis of glucose-stimulated Ca^2+^ traces revealed increases in both the percentage of correlated islet cell pairs (C) and correlative strength (*D* and *E*; *, *p* < 0.05; both Student's *t* test; *n* = 15 *Cre*^−^ and 17 *Cre*^+^ islets). Values represent mean ± S.D.

Although average islet Ca^2+^ responses to glucose were reduced by *Pax6* deletion (Fig. 6*A*), the responses of individual β cells were also altered. Specifically, islet β cells derived from *Cre*^−^ animals responded to glucose with a rapid and largely monophasic increase in free cytosolic Ca^2+^ that was synchronized across the islet (Fig. 7*A*, *top panel*, and supplemental movie 3). By contrast, β*Pax6*KO β cells displayed a delayed and oscillatory response, although one that was well coordinated between individual β cells (Fig. 7*A*, *bottom panel*, and supplemental Movie 4). Subjecting individual β cell responses to analysis packages that map cell-cell coordination (8, 44, 45), it was possible to show that the percentage of cells that responded to glucose was unchanged by *Pax6* deletion (Fig. 7*B*; 77.6 ± 5.39% *versus* 72.1 ± 4.81% for *Cre*^−^ *versus Cre*^+^, respectively; ns). However, the level of synchronicity between individual β cells tended to be higher in β*Pax6*KO islets, as shown by an increase in the percentage of significantly correlated cell pairs (Fig. 7*C*; 69.1 ± 7.40% *versus* 84.7 ± 3.79% for *Cre*^−^ *versus Cre*^+^, respectively; *p* = 0.06). Furthermore, the strength of β cell connections was greater in β*Pax6*KO islets, as shown by weighted graphs depicting the location and strength of connections between individual cells (Fig. 7, *D* and E; 0.42 ± 0.09 R *versus* 0.65 ± 0.04 R for *Cre*^−^ *versus Cre*^+^, respectively; *p* < 0.05). These changes were not associated with any alterations in the level of expression of the gap junction protein connexin 36, encoded by *Gjd2*, an important mediator of cell-cell interactions in the islet (7) (Fig. 3*I*).

## Discussion

This study sought to determine whether changes in cellular identity, described previously after *Pax6* inactivation in β cells (18, 19), are accompanied by significant changes in β cell function as well as the expression of key β cell genes involved in normal glucose sensing and insulin release. We also sought to determine the importance of *Pax6* in maintaining intercellular communication within the islet, a feature required for normal secretory responses to secretagogues that becomes defective in type 2 diabetes (21).

The use here of the efficient and selective Pdx1CreERT driver line (23, 24, 31) led to the more rapid (within days of tamoxifen treatment) and severe (>50 mm) hyperglycemia than observed previously after *Pax6* deletion either globally (18) or in β cells using the more promiscuous rat insulin promoter 2 (RIP2) Cre driver (19). Although limited recombination in the brain (chiefly in the basomedial hypothalamus) is also observed with Pdx1CreERT (22), deletion at extrapancreatic sites (19, 22, 46) or in islet non-β cells may contribute to the milder phenotypes observed previously with earlier *Cre*s. Although recombination with Pdx1CreERT has been reported previously to be almost completely confined to β cells (24), we did detect a small degree of deletion of PAX6 in α cells. This difference with respect to earlier findings may reflect the differing susceptibility to *Cre* action of *Lox*P sites at different genomic locations (47). Of note, glucagon mRNA levels were not different between WT and KO mice (Fig. 3*J* and supplemental Fig. S1*D*). On the other hand, we observed a substantial increase in ghrelin expression (Fig. 3*K* and supplemental Fig. S3*D*) after *Pax6* deletion, which may reflect a cell fate switch of β (or possibly α) cells toward an ϵ cell phenotype, as proposed previously (19).

We consequently chose to study islets from β*Pax6*KO mice at relatively early time points after deletion to minimize the exposure *in vivo* to high glucose concentrations. This was thought to be important given earlier (48) and more recent (49) reports that hyperglycemia itself leads to a loss of β cell identity. Although at the time point post-tamoxifen used in this study (8 days) a significant increase in fasting blood glucose was apparent (Fig. 2), the alterations in insulin and glucagon expression observed after *Pax6* deletion were markedly different in both extent (insulin) and direction (glucagon) from those reported previously in response to hyperglycemia induced by K_ATP_ channel activation in β cells (49). Moreover, in this study, islets were cultured at near normal (11 mm) glucose for 24–48 h after extraction, a maneuver expected to at least partially reverse any changes resulting from antecedent *in vivo* hyperglycemia. Finally, we observed no (qRT-PCR, Fig. 3*H*) or small (RNAseq, supplemental Fig. S1*B*) increases in *Ldha* expression, previously found to be reversibly up-regulated *in vivo* in islets by hyperglycemia (48). Thus, we conclude that the alterations in gene expression and function observed in this study are, at least in large part, a reflection of cell-autonomous actions of *Pax6* rather than hyperglycemia *per se*. Notably, the expression of several key β cell genes was affected by *Pax6* ablation, with substantial decreases in *Ins2*, *Slc30a8 (ZnT8*), *MafA*, *Slc2a2* (*Glut2*), and *Glp1r* mRNA levels (Fig. 3). Although future work will be required to determine whether this reflects direct or indirect regulation of these genes by PAX6, we note that *Gck* gene expression was increased (Fig. 3*F*), demonstrating a degree of selectivity in the regulation of β cell-enriched genes by PAX6.

Interestingly, we also demonstrate that, after *Pax6* inactivation, a significant increase occurs in the expression of members of the β cell disallowed gene family, notably *Slc16a1*/MCT-1 (27). As far as we are aware, this is the first time that *Pax6* has been shown to regulate members of this group. Thus, PAX6 controls β cell identity both through driving the expression of essential glucose-sensing genes and by *repressing* a gene whose presence is deleterious to β cell function (50). In this respect, the actions of PAX6 resemble those of RFX6, shown recently to be necessary for both normal β cell development (51) and function (34).

Although, and in agreement with previous studies, total islet insulin content was reduced by *Pax6* deletion by ∼60% (Fig. 2*F*), we were unable to detect any insulin secretion from *Pax6*-null islets in response to glucose. Interestingly, and although explored in a relatively small number of animals (*n* = 4/genotype), this was not associated with detectable decreases in overall β or α cell mass or in β/α cell ratio (Fig. 1, *G–I*). Deploying cell resolution imaging approaches, we demonstrate that this is associated with marked impairments in glucose-induced ATP/ADP increases, likely resulting from lowered Glut2 (*Slc2a2*) but not *Gck* expression (which was increased) and, possibly, increases in *Slc16a1*/MCT-1 levels, thus favoring anaerobic glycolysis. We also noted a substantial decrease in the expression of *G6pc2* (Fig. 3*N*), likely to potentiate the effects of increased *Gck* expression and thus to increase net flux from glucose to glucose-6P. Because flux control at the initial stages of glycolysis is achieved chiefly at the glucokinase step (1), the above observations suggest that increased flow through the latter reaction is not met by a proportional increase at later stages of glycolysis or in the oxidation of pyruvate and NADH by mitochondria. Future studies will be needed to explore these possibilities in detail. Of note, increased expression and release of ghrelin and binding to ghrelin receptors, which are enriched in δ cells (52), may also enhance somatostatin secretion to suppress insulin release.

Deletion of *Pax6* also drastically changed the profile of glucose-stimulated Ca^2+^ responses from an initial spike of Ca^2+^ activity followed by a sustained, oscillatory period (34, 53) to more delayed and burst-like properties reminiscent of those observed in α cells (Fig. 7*A*) (54). Interestingly, connectivity between cells was not significantly impaired by the loss of *Pax6*. Rather, we observed a tendency toward increased connectivity and a significant increase in the strength of connections between cells (correlation coefficient; Fig. 7, *D* and *E*) despite unchanged *Gjd2* expression (Fig. 3*I*). Thus, impaired β cell-β cell communication/connectivity does not drive altered glucose sensing in *Pax6*-null β cells. Importantly, our findings demonstrate that full β cell maturity is not a prerequisite for the formation of a functional interconnected network of cells. Indeed, we have recently shown the existence of a small population of immature β cells that express high levels of glucokinase and that dictate islet responses to glucose (55). Whether and how the activity of these putative “hub” or “pacemaker” cells within the islet is affected by *Pax6* deletion will be interesting to establish. Of note, elevated *Gck* expression, alongside substantially lower *Pdx1* and *Nkx6.1* expression, in β*Pax6*KO islets is a feature characteristic of previously described islet hub cells (55). These and other changes, including in ion channel expression (RNAseq; “Results” and data not shown) and glucose sensitivity, may thus contribute to the enhanced Ca^2+^ dynamics and connectivity observed after *Pax6* deletion.

Perhaps surprisingly, the partial defects in metabolic glucose signaling observed above (*i.e.* ATP/ADP and Ca^2+^ changes) were associated with an apparently complete loss of glucose-stimulated insulin secretion, assessed *in vitro*. This loss of insulin release was less marked *in vivo*, possibly reflecting alternative mechanisms for stimulating secretion in the living animal (*e.g.* actions of circulating amino acids or fatty acids, hormonal and neural inputs into the islet, etc.) (1). Nevertheless, the results obtained with isolated islets would appear to indicate that PAX6 is also important in the β cell for K_ATP_ channel- and Ca^2+^-independent mechanisms of glucose-stimulated insulin secretion (56). Whether enhanced β cell-β cell coupling can, in some settings, also be deleterious for the normal tight regulation of insulin secretion is a possibility that also merits investigation.

While the current study was under revision, a report (57) appeared using a similar approach but an alternative *Cre* (MIP.CreERT), based on the mouse insulin 1 promoter, to delete *Pax6* in adult β cells. Importantly, Swisa *et al.* (57) also identified, by gene ontology analysis, gene classes that may contribute to the enhanced connectivity observed in this report, including ion channels and Ca^2+^ channels. As observed here, the phenotype of β *Pax6*-null mice was remarkably severe and associated with ketosis and marked changes in secretory granule structure observed at the ultrastructural level by electron microscopy. Thus, atypical granules, lacking a classical “halo” around the dense core, were frequently seen in null mouse islets. Importantly, lineage tracing work in the report by Swisa *et al.* (57) also demonstrated that “reprogrammed” cells, expressing ghrelin, were derived from β cells. Finally, it was shown by ChIP sequencing that PAX6 binds directly to sequences in target genes to affect their repression or activation. Importantly, however, the above study did not explore the regulation of insulin secretion or metabolic glucose signaling in *Pax6*-deleted β cells, as described in this report.

As discussed above and elsewhere (58), use of the MIP.CreERT deleter strain may, however, be complicated by the concomitant overexpression of human growth hormone. Reassuringly, the strong similarities between the findings of our own study and those described in Ref. 57 indicate that the changes in each are chiefly attributable to altered *Pax6* expression.

## Experimental procedures

### Generation of βPax6KO mice

All animal procedures were approved by the Home Office according to the Animals (Scientific Procedures) Act 1986 of the United Kingdom (PPL 70/7349).

Mice homozygous for the *floxed* allele of *Pax6* (*Pax6*^fl/fl^), kindly provided by Prof. David Price (University of Edinburgh) (59), were crossed to a tamoxifen-regulated deleter strain provided by Dr. Doug Melton (Harvard Medical School) in which *Cre* recombinase is expressed under the control of the Pdx1 promoter (Pdx1CreERT) (23). The breeding strategy ensured that 50% of the offspring inherited the *Cre* transgene (*Cre*^+^, β*Pax6*KO; Pdx1CreERT^+/−^::Pax6^fl/fl^), the remainder serving as littermate controls (*Cre*^−^, Pdx1CreERT^−/−^::Pax6^fl/fl^). Gene deletion was achieved by five intraperitoneal tamoxifen injections (2 mg), administered daily to 8-week-old animals of both genotypes. Pdx1CreERT^+/−^ mice displayed no evident metabolic abnormalities.

### Insulin tolerance tests

Bovine insulin (0.75 units/kg body weight, Sigma) was injected into the abdomen of mice fasted for 5 h. Blood glucose measurements were taken at 0, 15, 30, and 60 min using an automatic glucometer (Accu-Chek).

### Plasma glucose and insulin measurements

Fed mice were culled, and blood was collected into EDTA-coated tubes (Sarstedt, Beaumont Leys, UK). Glucose measurements were determined using an automatic glucometer (Accu-Chek). For insulin measurements, plasma was separated by centrifugation at 13,200 rpm for 20 min. 5 μl of blood plasma was used to measure insulin levels using an ultrasensitive mouse insulin ELISA kit (CrystalChem). For total islet insulin measurements, five islets were lysed by sonication in acidified ethanol (1.5% HCl (v/v), 75% ethanol (v/v), and 0. 1% Triton X-100 (v/v)). Insulin concentration was determined using a HTRF-based assay kit (CisBio).

### qRT-PCR

RNA was extracted from isolated islets using TRIzol reagent (Invitrogen) and reverse-transcribed using a high-capacity reverse transcription kit (Invitrogen). Relative gene expression was assessed using SYBR Green (Invitrogen), and expression of each gene was normalized and expressed as *n*-fold change in mRNA expression *versus* Cre^−^, calculated using the 2^ΔΔCt^ method (60).

### Massive parallel sequencing of RNA (RNAseq)

Total RNA was extracted with TRIzol from islets isolated from five knock-out and four control mice. Polyadenylated transcripts were selected during the preparation of paired-end, directional RNAseq libraries. Libraries were sequenced on an Illumina HiSeq 2000 machine. The quality of the sequenced libraries was assessed using fastQC. Reads were mapped to the Grc38m assembly using HiSat2. Annotated transcripts were quantified using featureCounts, and differentially expressed genes were identified with DESeq2. All genes were ranked by -fold change, and the resultant list was used for gene set enrichment analysis. Gene set enrichment analysis (GSEA) was performed essentially as described previously (61), and we used the Broad Institute GSEA tool for analysis of preranked lists with a custom-constructed gene set combining the disallowed genes identified in Ref. 25, 26.

### RNAseq accession codes

Raw sequence data for RNAseq will be made available via deposition to ArrayExpress.

### Immunocytochemistry

Pancreata were extracted and fixed in 10% (v/v) formalin, transferred into a tissue-processing cassette, and embedded in paraffin wax within 24 h of being harvested from the mouse. Pancreatic sections were cut at 5 μm onto Superfrost slides. Sections were dewaxed by immersion in Histoclear (Sigma-Aldrich, Dorset, UK) for 20 min before rehydration. Antigen retrieval was performed by boiling slides in citrate buffer (Vector Laboratories, Peterborough, UK), washed before blocking with 3% (v/v) goat/donkey serum (Dako, Cambridgshire, UK) in 2% BSA with 0.1% (v/v) Triton X-100 for 1 h at room temperature. Slides were then washed before staining with primary antibody (guinea pig anti-insulin (1:200, Dako), mouse anti-glucagon (1:1000, Sigma-Aldrich), and rabbit anti-chromagranin A (1:200, Abcam). Slides were incubated in primary antibody overnight at 4 °C. Revelation was performed using a combination of the following secondary antibodies: donkey anti-rabbit 488, goat anti-guinea pig 643, and goat anti-mouse 568 (all 1:500, Invitrogen). Slides were mounted using Vectashield mounting medium containing DAPI (Vector Laboratories), and a 24-mm coverslip was placed over the slice. Slides were visualized by fluorescence microscopy using a Zeiss Axio Observer inverted wide-field microscope with LED illumination running Zen acquisition software. Quantification of the immunopositive area was achieved using ImageJ analysis software using a macro established in-house. For quantification of β and α cell mass, slides were visualized using an Axiovert 200 M microscope (Zeiss) with Alexa Fluor 488 goat anti-guinea pig IgG and Alexa Fluor 568 goat anti-mouse IgG (Invitrogen) using an Axiovert 200 M microscope (Zeiss, Welwyn Garden City, UK) at the Facility for Imaging by Light Microscopy (Imperial College London).

To stain PAX6, an alternative protocol was used based on a modification of the protocol described in Ref. 62. The main adaptions included permeabilization with methanol (100%, −20 °C, 3 min), more vigorous antigen retrieval via boiling in citrate-based antigen unmasking solution (Vector Laboratories, 15 min), enhanced blocking with 1% (w/v) skimmed milk (1.5 h), and incubation with anti-glucagon antibody (1:500) alone for 2.5 h before overnight incubation with the remaining two primary antibodies (anti-PAX6, Biolegend, 1:70 and anti-insulin, 1:200) and a longer incubation with secondary antibody (2 h). Image acquisition was performed with a Zeiss Axio Observer inverted microscope fitted a Hamamatsu Flash4 camera. The filters used were as follows: blue (365-nm LED; excitation, 377/50; emission, 477/60), green (470-nm LED; excitation, 472/30; emission, 520/35), red (540- to 580-nm LED; excitation, 534/20; emission, 572/28), and far red (625-nm LED; excitation, 631/22; emission, 688/20). Negative controls to assess nonspecific binding were performed by omitting incubation with primary antibodies.

### Western blotting

Whole-islet extracts were separated using a 10% acrylamide gel and blotted onto a polyvinylidene difluoride membrane. Following blocking for 60 min with 5% (w/v) skimmed milk in TBST (10 mm Tris base (pH 7.6), 150 mm NaCl, and 0.1% Tween 20), membranes were incubated overnight at 4 °C with an antibody against Pax6 (1:300, Biolegend). The membrane was washed three times (5 min each) with TBST before incubation of the secondary anti-rabbit HRP antibody (1:5000) for 1 h. An ECL system (GE Healthcare) was used for activation according to the guidelines of the manufacturer. The film was then exposed for 3 s and developed on a Xograph Compact X5 film processor. The membrane was then stripped for incubation with rabbit anti-tubulin (1:5000) as a loading control and re-exposed as above (5 s).

### Ca^2+^ imaging and connectivity analysis

Isolated islets were incubated (37 °C, 95% O_2_, 5% CO_2_) for 1 h in Fluo-2/AM (10 μm; Teflabs, Austin, TX) diluted in a HEPES-bicarbonate buffer solution (120 mm NaCl, 4.8 mm KCl, 1.25 mm NaH_2_PO_4_, 24 mm NaHCO_3_, 2.5 mm CaCl_2_, 1.2 mm MgCl_2_, 10 mm HEPES, and 3 mm d-glucose; all from Sigma). Functional multicellular Ca^2+^ imaging was achieved using a Nipkow spinning disk head, allowing rapid scanning of islet areas for long periods of time with minimal phototoxicity. A solid-state laser (CrystaLaser) controlled by a laser merge module (Spectral Applied Physics) provided wavelengths of 491 nm to excite Fluo-2 (rate, 0.5 Hz; exposure time, 600 ms). Emitted light was filtered at 525/50 nm, and images were captured with a highly sensitive 16-bit, 512 × 512 pixel back-illuminated EM-CCD camera (ImageEM 9100-13, Hamamatsu). Volocity^TM^ software (PerkinElmer Life Sciences) provided the user interface. During recordings, islets were maintained at 35 °C to 36 °C and continuously irrigated with bicarbonate buffer aerated with 95% O_2_, 5% CO_2_. Connectivity analysis was performed as described previously (8, 45).

### Measurement of insulin secretion in vitro

Insulin secretion was measured in batch incubations essentially as described previously (63). In brief, batches of 20 islets from βPax6 KO or control mice were subjected to glucose-stimulated insulin secretion assay 1 week after tamoxifen administration (*n* = 3 animals/genotype). Islets were incubated for 30 min sequentially in 3 mm and then 17 mm glucose. Islets were then lysed in lysis buffer (50 mm TrisCl (pH 8.0), 150 mm NaCl, 1% v/v IGEPAL-C130 (Sigma-Aldrich)), and nuclei were pelleted. The insulin content of secreted and total fractions was determined by HTRF assay and normalized to the DNA content of the nuclear fraction, assayed using CyQuant fluorescent dye.

### ATP/ADP imaging

Islets were infected with an adenovirus containing cDNA encoding the ATP sensor Perceval (41) for 48 h to measure intracellular ATP dynamics. Excitation of the fluorescent probe was achieved using a 491-nm laser (Spectral Applied Physics; rate, 0.2 Hz; exposure time, 600 ms). Emitted light was filtered at 525/50 nm, and images were captured as for Ca^2+^ imaging.

### Total internal reflectance of fluorescence (TIRF) analysis

Islets were infected with an adenovirus encoding neuropeptide Y-venus as described previously (64). Islets were dissociated as described previously (65), and the liberated cells were allowed to adhere to poly-l-lysine-treated sterile glass slides for 24 h post-transfection. Cells were then incubated for 1 h in HEPES bicarbonate buffer (see Ca^2+^ imaging analysis) containing 3 mm glucose at 37 °C. Fixation was performed by incubating the slides for 1 h at room temperature in PBS supplemented with 4% (v/v) paraformaldehyde.

Imaging was performed as described previously (66) using a Nikon Eclipse Ti microscope equipped with a ×100/1.49 numerical aperture (NA) TIRF objective, a TIRF/fluorescence recovery after photobleaching (FRAP) iLas2 module o control laser angle (Roper Scientific), and a Quad Band TIRF filter cube (TRF89902, Chroma). Neuropeptide Y-venus, localized in insulin-secreting vesicles (67), was excited using a 488-nm laser line, and images were acquired with an ORCA-Flash 4.0 camera (Hamamatsu), and Metamorph software (Molecular Devices) was used for data capture. The laser angle was selected for an imaged section thickness of 150–180 nm. Images were analyzed on ImageJ using a home-made macro. The script of the macro is available upon request.

### Statistical analysis

Statistical significance was assessed using Student's *t* test. Two-way analysis of variance (with Bonferroni or Sidak multiple comparisons test) was used to examine the effect of multiple variables. Statistical analyses were performed using GraphPad Prism 7.0, ImageJ, and IgorPro. Values represented are the mean ± S.D.

## Author contributions

R. K. M., M. S. N. T., P. C., R. M. C., T. J. P., I. L., and R. C. performed all experimental work and wrote the manuscript. G. A. R. supervised the work and wrote the manuscript. D. J. H. co-supervised the work and edited the manuscript. G. A. R. is the guarantor of the manuscript.

## Supplementary Material

Supplemental Data

## Footnotes

4 The abbreviations used are:

ns: not significant
TIRF: total internal reflectance of fluorescence
qRT-PCR: quantitative real-time PCR
RNAseq: RNA sequencing
GSEA: gene set enrichment analysis
AU: arbitrary units
HTRF: homogeneous time resolved fluorescence
LED: light-emitting diode.
