# Supplementary material for: The transcription factor Pax6 is required for pancreatic β cell identity, glucose-regulated ATP synthesis, and Ca2+ dynamics in adult mice
Source: J Biol Chem. 2017 Apr 4;292(21):8892–906. doi: 10.1074/jbc.M117.784629 (PMC5448123; doi:10.1074/jbc.M117.784629)
Supplement: Supplemental Data [file supp_292_21_8892__index.html]

The transcription factor Pax6 is required for pancreatic β cell identity, glucose-regulated ATP synthesis and Ca2+ dynamics in adult mice — The transcription factor Pax6 is required for pancreatic β cell identity, glucose-regulated ATP synthesis, and Ca2+ dynamics in adult mice — Pax6 controls functional β cell identity — Supplemental Data 

# The transcription factor *Pax6* is required for pancreatic β cell identity, glucose-regulated ATP synthesis, and Ca2+ dynamics in adult mice

## Supplemental Data

- Supplemental Movie 1 (.avi, 1.9 MB) - Gucose-stimulated ATP dynamics, control islet
- Supplemental Figure S1 (.pdf, 211 KB) - RNA seq analysis
- Sipplemental movie 2 (.avi, 2.5 MB) - ATP dynamics; KO islet
- Supplemental movie 3 (.avi, 2.5 MB) - Glucose-stimulated Ca2+ dynamics, control islet
- Supplemental movie 4 (.avi, 2.0 MB) - Glucose-stimulated Ca2+ dynamics, KO islet
