# Supplementary material for: The transcription factor Pax6 is required for pancreatic β cell identity, glucose-regulated ATP synthesis, and Ca2+ dynamics in adult mice
Source: J Biol Chem. 2017 Apr 4;292(21):8892–906. doi: 10.1074/jbc.M117.784629 (PMC5448123; doi:10.1074/jbc.M117.784629)
Supplement: Supplemental Data [file 10.1074_M117.784629_jbc.M117.784629-2.pdf]

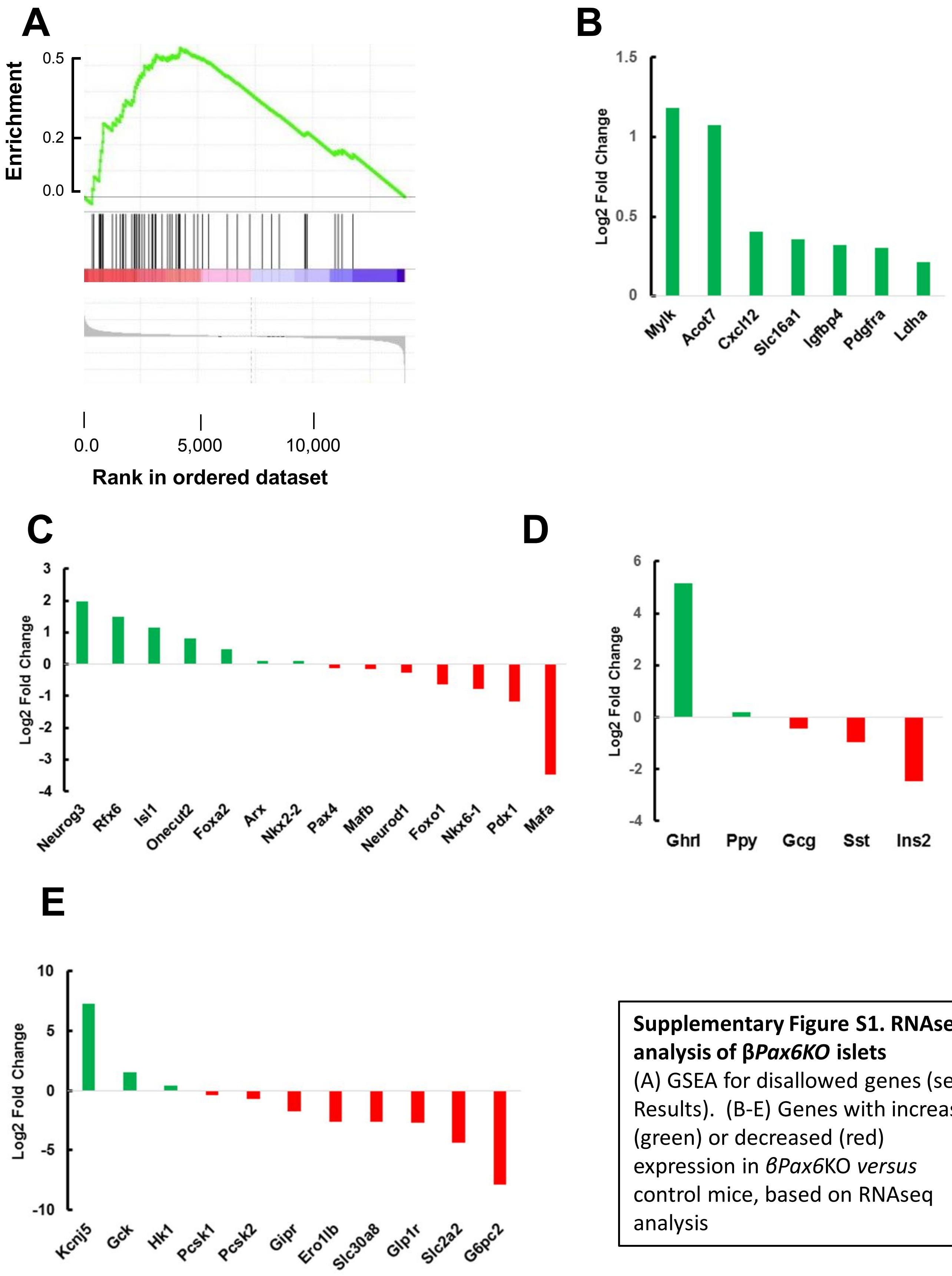

Supplementary Figure S1. RNAseq analysis of β*Pax6KO* islets
(A) GSEA for disallowed genes (see Results). (B-E) Genes with increased (green) or decreased (red) expression in *βPax6*KO *versus* control mice, based on RNAseq analysis
